# Enhanced Stability, Superior Anti-Corrosive, and Tribological Performance of Al_2_O_3_ Water-based Nanofluid Lubricants with Tannic Acid and Carboxymethyl Cellulose over SDBS as Surfactant

**DOI:** 10.1038/s41598-024-59010-w

**Published:** 2024-04-22

**Authors:** Dieter Rahmadiawan, Shih-Chen Shi

**Affiliations:** Department of Mechanical Engineering, National Cheng Kung University (NCKU), Tainan, Taiwan

**Keywords:** Alumunium oxide, Nanofluid, Tannin acid, Carboxymethyl cellulose, SDBS, Synthesis and processing, Mechanical engineering, Nanoparticles

## Abstract

In this research work, the stability, tribological, and corrosion properties of a water-based Al_2_O_3_ nanofluid (0.5 wt%) formulated with tannin acid (TA) and carboxymethyl cellulose (CMC) as dispersants or surfactants were investigated. For comparative purposes, sodium dodecylbenzene sulfonate (SDBS) was also incorporated. The stability of the nanofluid was assessed through zeta potential measurements and photo-capturing, revealing the effectiveness of TA and CMC in preventing nanoparticle agglomeration. Tribological properties were examined using a pin-on-disk apparatus, highlighting the tribofilm of Al_2_O_3_ that enhanced lubricating properties of the nanofluid by the SEM, resulting in reduced friction and wear of the contacting surfaces. Sample with the addition of both TA and CMC exhibited the best tribological performance, with a ~ 20% reduction in the friction coefficient and a 59% improvement in wear rate compared to neat nanofluid without TA and CMC_._ Additionally, the corrosion resistance of the nanofluids were evaluated via weight loss and electrochemical impedance spectroscopy. The nanofluid sample containing both TA and CMC exhibited the lowest corrosion rate, with 97.6% improvement compared to sample without them. This study provides valuable insights into the potential applications of TA and CMC-based Al_2_O_3_ nanofluids as effective and environmentally friendly solutions for coolant or lubrication in cutting processes.

## Introduction

Water-based nanofluids have attracted significant attention recently due to their high potential to enhance thermal, mechanical, and chemical properties and suitable application as lubrication fluid and coolant^1,2^. Among the numerous nanoparticles explored for water-based nanofluid applications, alumunium oxide (Al_2_O_3_) nanoparticles is a promising candidate due to their excellent thermal conductivity, tribological properties, corrosion resistance, and low cost compared to other advanced nanoparticles^3–5^. Wang et al. reported that water-based Al_2_O_3_ nanofluid could reduce the wear rate by ~ 42% compared to base fluid^6^. It has also been reported that Al_2_O_3_ can significantly improve the corrosion inhibition of brass. This effect is attributed to the capability of Al_2_O_3_ nanoparticles to form a protective film on the brass surface^7^.

However, exploring the full potential of Al_2_O_3_ water-based nanofluids requires overcoming challenges related to achieving excellent dispersion or stability of the nanoparticles^8^. Two common methods to improve nanofluid stabilization are by using sonicator or surfactant. Sonication involves subject ting the nanofluid to high-frequency sound waves, which create intense cavitation and shear forces^9^. These forces disperse and break down agglomerated particles into smaller, more uniform sizes, which enhance the stability of the nanofluid. The surfactant method, on the other hand, is coorporating surfactant molecules into the nanofluid. Due to its nature, these molecules will surround nanoparticle’s surfaces and form a protective layer which reduce inter-particle forces^10^.

Sodium Dodecyl Benzene Sulfonate (SDBS) is a common surfactant for improving the Al_2_O_3_ water-based nanofluid^11^. However, excessive use of SDBS for nanofluid stabilization is not recommended^12^. A high concentration can cause surfactant to be over-adsorption, which could destabilize the nanofluid, while too little might not provide enough coverage to stabilize the nanoparticles^8^. Despite their potential, the practical application of nanofluids using SDBS faces critical challenges, particularly in long terms stability. Moreover, if the nanofluid is applied as lubricants, several properties such as corrosion resistance and tribological properties need to be considered. This research gap underscores the need for an approach to nanofluid development, one that not only focuses on stability performance gains but also considers tribological and corrosion resistance.

Tannin acid (TA), derived from plant sources such as fruits, leaves, and bark, is a polyphenolic compound known for its excellent complexation and adsorption abilities^13^. It has also been found that TA could generate steric repulsion to stabilize nanoparticles due to its reducibility and coating property^14,15^. Furthermore, TA’s hydrophilic nature and the presence of phenolic hydroxyl groups (–OH), which have a strong affinity for metal surfaces, make it an attractive candidate for improving corrosion resistance and stability^16^.

Carboxymethyl cellulose (CMC), on the other hand, is a water-soluble derivative of cellulose with versatile applications, including its use as a lubricant additive. This is attributed to its properties that promote thickening ability^17,18^. It is environmentally friendly and has hydrophilic structure, making it an effective dispersant for water-based nanofluid. The long chains of CMC can impart electrostatic repulsion through the negative charges on its molecular chains^19,20^. Previous work has demonstrated that the addition of CMC improves the stability of Al_2_O_3_ nanofluids due to its electrostatic repulsion, which counteracts the van der Waals forces^21^.

This work hypothesizes that introducing TA and CMC into 0.5 wt% Al_2_O_3_ nanofluids will enhance the stabilization of Al_2_O_3_ nanoparticles, leading to improved dispersion, tribological performance, and increased corrosion resistance. The presence of tannic acid (TA) can offer steric repulsion stabilization and corrosion protection. This is attributed to its polyphenolic compound, which can adsorb onto the metal surface and react with metal ions. On the other hand, CMC will provide electrosteric repulsion, preventing attractive force between Al_2_O_3_ nanoparticles. The dual action of TA and CMC can enhance the dispersion of Al_2_O_3_ nanoparticles in the base fluid through electrostearic repulsion^22^. Moreover, not only acting as a stabilizer, but CMC also has the potential to increase water viscosity, resulting in a thicker boundary film, which reduces the friction between the rubbed surface^18^.

The stability, tribological behavior, and corrosion resistance of 0.5 wt% Al_2_O_3_ water-based nanofluids with TA and CMC as additives will be explored for the first time in this work. As the author is aware, the incorporation of TA and CMC to improve the stability of Al_2_O_3_ nanofluids, leading to enhancement in tribocorrosion performance, has not yet been investigated. The scope of this study is limited to water-based nanofluids containing Al_2_O_3_ nanoparticles, with a particular focus on their application as lubricants. By integrating a combination of TA and CMC, the research aims to provide comprehensive insights into the effect of these additions on nanofluid properties.

The study endeavors to contribute to the field of nanofluids by incorporating natural-based additives, thereby facilitating the development and application of nanofluid lubricants. These lubricants aim to be not only effective but also environmentally responsible. Ultimately, the findings of this research have the potential to pave the way for the development and optimization of water-based Al_2_O_3_ nanofluid. These novel additives could offer superior performance and environmentally friendly properties, making them suitable for use as coolants or lubricants in cutting processes.

## Materials and methods

### Materials

Spherical-shaped Al_2_O_3_ with a particle size of 300 nm and purity of 99% was purchased from Ultimate Materials Technology Co., Ltd (Hsinchu, Taiwan). CMC (pH 6.0–8.0) was purchased from Showa Chemical Co., Ltd (Tokyo, Japan). TA was purchased from Daejung Co. (South Korea). Sodium dodecylbenzenesulfonate (SDBS) was purchased from AK Scientific (Union City, USA).

### Nanofluid preparation

**Figure 1:**
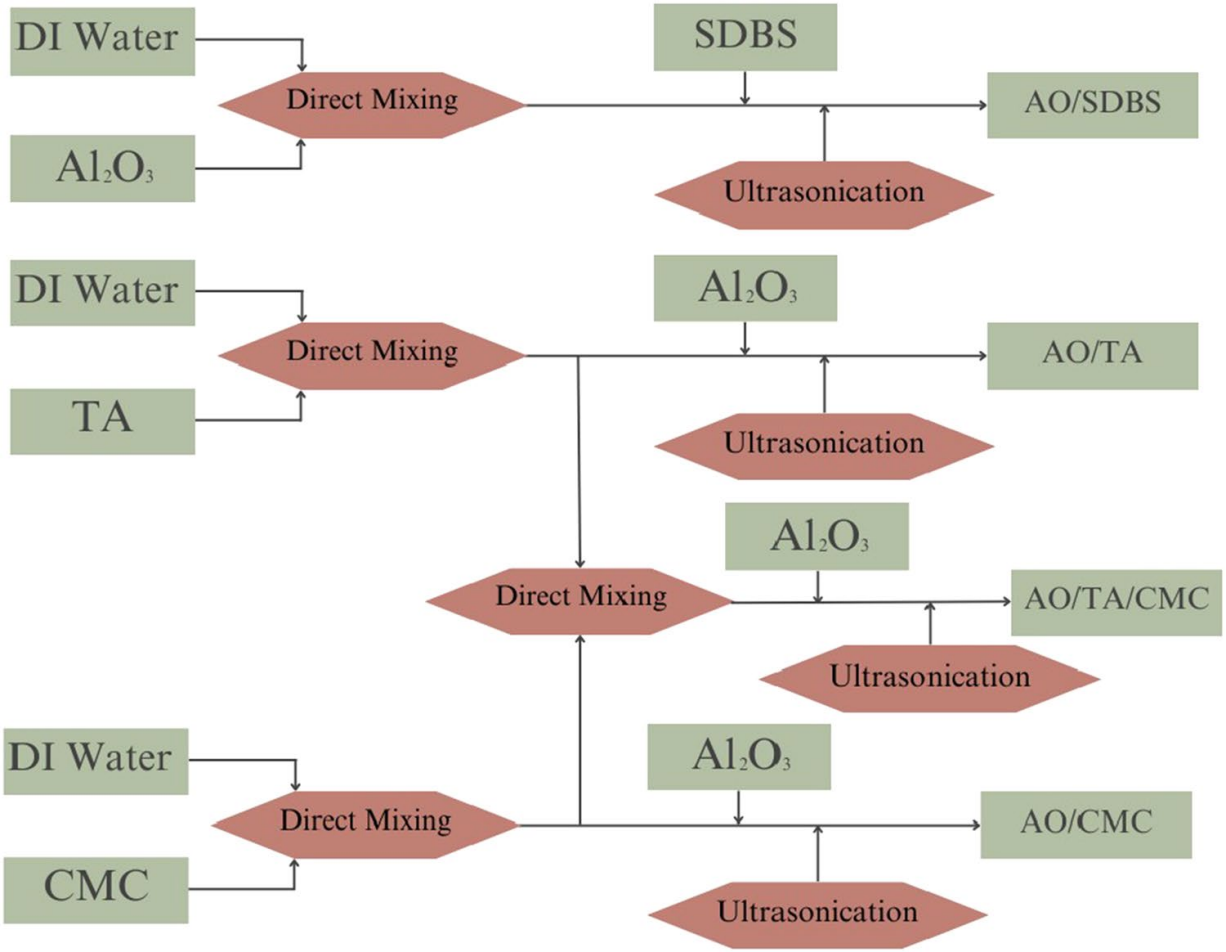
Preparation procedure of the water-based nanofluid.

Table 1 shows the composition of each nanofluid sample. To prepare the AO/SDBS sample, deionized water and Al_2_O_3_ (0.5 wt%) were stirred for 30 min, followed by the addition of SDBS (0.15 wt%). Figure 1 shows the preparation procedure for the AO/TA/CMC nanofluid.The AO/TA/CMC nanofluid preparation involved several sequential steps. Firstly, deionized water was combined with 0.15 wt% of TA, stirred at 80 °C for 30 min, ensuring complete dissolution. Subsequently, Al_2_O_3_ nanoparticles (0.5 wt%) were gradually introduced into the TA solution, followed by the addition of SDBS (0.15 wt%). Concurrently, a separate solution of CMC (0.15 wt%) was prepared by dissolving it in deionized water. The final step involved slowly combining the CMC solution with the mixture of water, Al_2_O_3_, and tannic acid, and finally, sonicated it with a sonicator for 15 min (600 W). The temperature of the sample was carefully monitored so that it did not exceed 60 °C. The concentrations of all components were determined based on optimal values reported in the existing literature^11,21,23^.

**Table 1:** Composition of nanofluid lubricant samples.

Sample	Concentration (wt%)				
	DI Water	Al_2_O_3_	TA	SDBS	CMC
AO/SDBS	99.35	0.5	0	0.15	0
AO/TA	99.35	0.5	0.15	0	0
AO/CMC	99.35	0.5	0	0	0.15
AO/TA/CMC	99.20	0.5	0.15	0	0.15

### Nanofluid characterization

The particle size of the Al_2_O_3_ nanoparticles was characterized using scanning electron microscopy (SEM) (SU-5000, Hitachi, Japan). Approximately 0.1 wt% of the Al_2_O_3_ nanofluid was poured onto the surface of a disk. This disk was then placed in a drying oven for several days until all the liquid had evaporated. Then, the dried surface was examined using SEM to accurately determine the particle size of the Al_2_O_3_ nanoparticles. To confirm the chemical composition of each nanofluid sample, the Fourier transform infrared spectroscopy (FTIR)(Nicolet iS5, ThermoFisher Scientific, USA) test was performed.

### Nanofluid stability (dynamic light scattering and photo capturing)

The stability of each nanofluid was assessed both quantitatively and qualitatively. Dynamic light scattering (Delsa Nano C, Beckman Coulter, USA) was employed to investigate the zeta potential of the nanofluids. Samples were photographed immediately after preparation, after 24 h, after 2, 4, 8, and 15 days.

### Tribological performance test

The tribological performance was investigated using a ball-on-disk tribometer (POD-FM406-10NT, Fu Li Fong Precision Machine, Kaohsiung, Taiwan) with a load of 16 N, disk speed of 0.1 m/s, rotation speed of 191 rpm, and sliding distance of 90 m. The disk, with a diameter of 24 mm and a thickness of 10 mm, was made of a Cu–Zn alloy (60% Cu–40% Zn), while the ball material was chrome steel. The hardness and surface roughness of the disk were 120 HV and 3.7 μm, respectively. The Cu–Zn alloy was specifically chosen for the disk to facilitate the detection of Al_2_O_3_ particles within the wear scar. The test was carried out in accordance to ASTM G-99^24^. Following the test, the area of the disk scar (depth x length) was examined using a 3D laser scanning microscope. SEM (SU-5000, Hitachi, Japan) with EDS (EDX, JSM-7800 F, JEOL) was employed to investigate and analyze the behavior of the nanofluid inside the scar of the disk.

### Anti-corrosion performance test

The corrosion performance of the Cu–Zn disk was investigated using electrochemical impedance spectroscopy (EIS, Jiehan, HIOKI 3533-05) and the weight loss method. The Cu–Zn alloy demonstrates a robust reaction with TA, thereby allowing for a detailed analysis of the corrosion inhibiting effects of this additives^25^. For the EIS method, a liquid composed of 96.5 wt% water and 3.5 wt% NaCl served as the electrolyte. The test was carried out over a frequency range from 100 kHz to 10 mHz, with 5 points recorded per decade. Before conducting the test, the Cu–Zn disk was coated with nanofluid by dipping it onto the surface of the disk and then left for 3 days. Afterwards, the particles in the nanofluid will adhere to the surface of the disk and will form a film. The equivalent circuit model for EIS is consistent with previous studies that analyze protective films or coatings^26^.

The weight loss method was conducted according to the literature^27^. The disk was immersed in a nanofluid with an addition of 3.5 wt% NaCl to speed up the corrosion process. It was immersed at a temperature of 50 °C for 3 days. The weights of the disk before and after immersion were recorded to determine the weight loss. The weight loss corrosion rate was calculated using the following Equation^16^:

$$v= \frac{({W}_{a}-{W}_{b})}{St}$$

where $v$ is the weight loss corrosion rate (g/(m^2^ h), ${W}_{a}$ is the weight of specimens before immersion (g), ${W}_{b}$ is the weight of specimens after immersion without corrosion products (g), $S$ is the surface area of the disk (m^2^), and $t$ is the total immersion time (h).

## Results and discussions

### Morphology of Al_2_O_3_ nanoparticle

**Figure 2:**
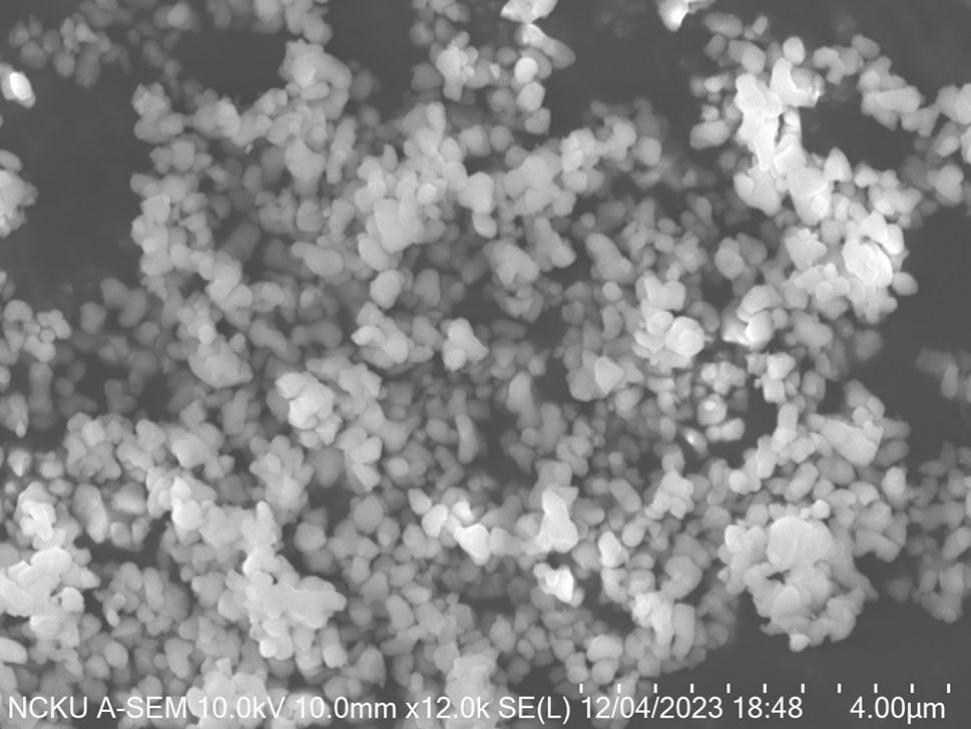
SEM image of Al_2_O_3_ nanoparticle.

The SEM images of the Al_2_O_3_ nanoparticles are shown in Fig. 2. It can be seen that the particle exhibits a well-defined spherical shape, as evidenced by the SEM images obtained at a magnification sufficient to discern individual particles. The average diameter of these nanoparticles is measured to be approximately 300 nm, indicating a high degree of uniformity in size across the sample. The choice of a 300 nm size for Al_2_O_3_ is aligning with several tribological considerations that enhance their effectiveness on metal surfaces. While smaller nanoparticles (below 100 nm) offer certain advantages like higher surface area and more stable if dispersed in fluid, their effectiveness as lubricant additives might be different^28^. Smaller nanoparticles may lack the load-bearing capacity needed for effective protection under high-stress conditions. Their diminutive size might lead to insufficient film formation, limiting their ability to withstand heavy loads and prevent metal-to-metal contact. This statement is supported by the literature review, which indicates that nanoparticles with a size of 300 nm exhibit a coefficient of friction (CoF) value lower than 0.1, similar to that of smaller nanoparticles^29^. Moreover, smaller nanoparticles are relatively more expensive, as producing small particles is more time-consuming compared to larger ones.

### FTIR analysis

**Figure 3:**
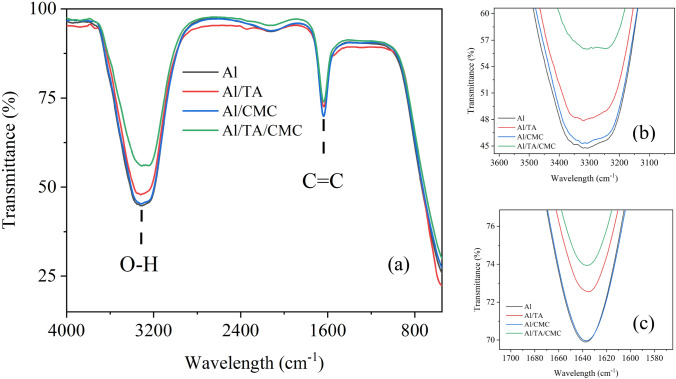
FTIR full spectra (**a**), zoom between 3600–3100 (**b**), and 1700–1580 cm^-1^ of all nanofluid samples.

Figure 3 displays the FTIR spectra from 4000 to 500 cm^−1^ for all samples. It can be seen that the main peak for all nanofluids were observed at 3300 and 1600 cm^−1^. These peaks are corresponding to stretching vibration of the O–H (hydroxyl) and C=C bond, accordingly^30,31^. After the addition of TA or CMC, the intensity of the 3300 and 1600 cm^−1^ peak was notably reduced. At the 3300 cm^−1^ peak, the intensity for the AO/TA/CMC sample decreased by approximately 20% compared to the AO/SDBS sample. This indicates that the addition of TA or CMC, or both, led to lower intensities at these wavelengths. Both CMC and TA has hydroxyl groups (OH), which can potentially form hydrogen bonds with water molecules^32^.

The formation of hydrogen bonds can alter the vibrational characteristics of the OH bonds, leading to changes in the observed FTIR spectrum^33^. The OH stretching vibrations may be affected, resulting in a reduction in intensity. Additionally, this could also possibly be due to electrostatic interactions.

### Nanofluid stability analysis

#### Photo capturing method

**Figure 4:**
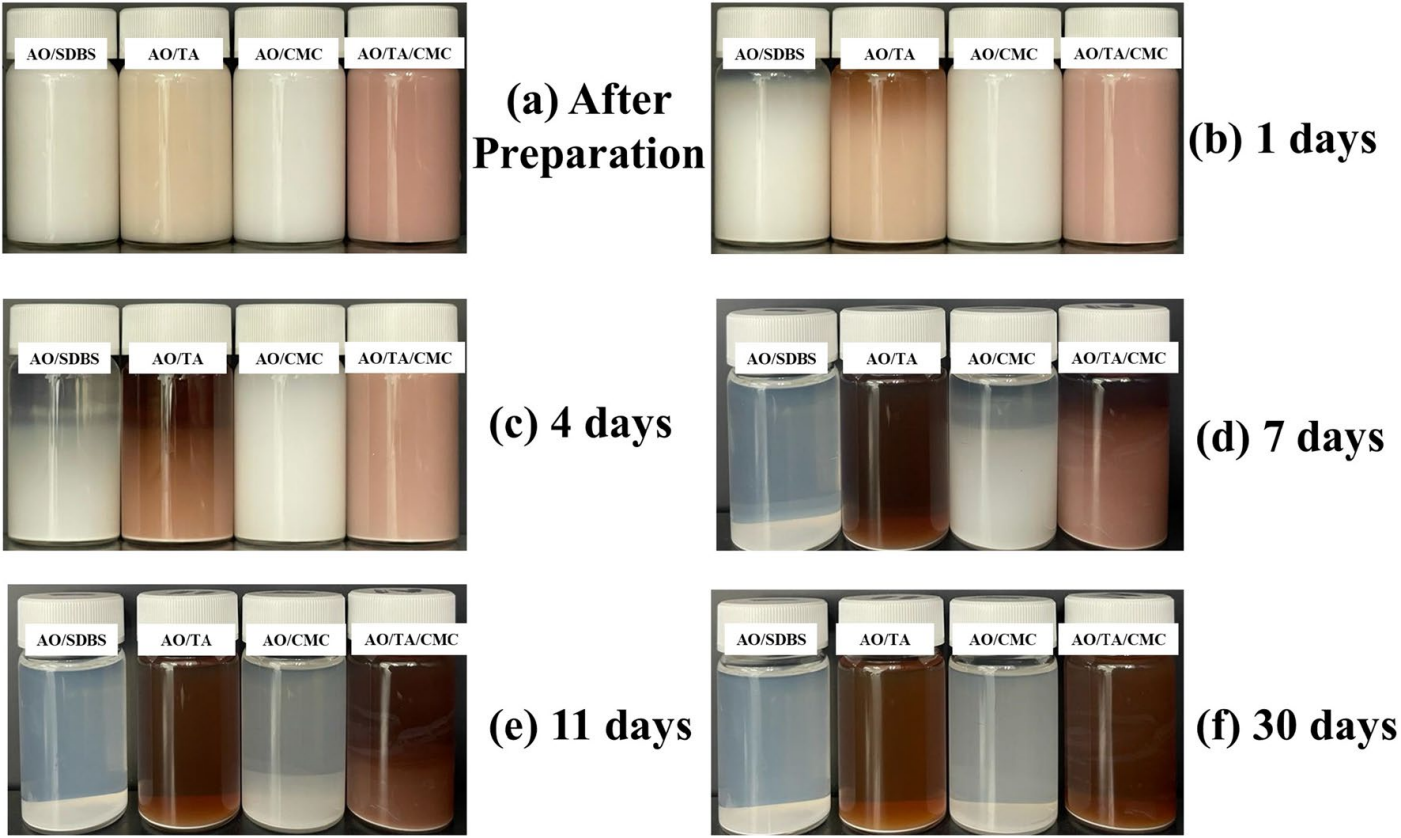
Photo capturing stability observation of the nanofluids after preparation (**a**), after 24 (**b**), 96 (**c**), 168 (**d**), 264 (**e**), and 720 h.

Figure 4 shows the photo capturing of nanofluid samples, indicating their stability after preparation and after being left for 30 days. On day 1 (Fig. 4b), or after preparation, all samples exhibit good stability visually. However, after being left for a day, sedimentation begins to be observed for the AO and AO/TA samples. It becomes more visible on day 4 (Fig. 4c), with approximately 50% sedimentation. The samples were fully sedimented by day 7 (Fig. 4d). On the other hand, samples with CMC show relatively good stability.

Until day 4, the AO/CMC and AO/TA/CMC samples can prevent sedimentation more effectively compared to those without CMC. The samples maintain stability at around 80% until day 7. After that, the sample started to settle, and they were almost fully sedimented by day 11 (Fig. 4e). On the other hand, in Fig. 4f, the sample with CMC needs 30 days for nanoparticles to totally sediment, indicating its stability is far better than the one with SDBS or TA.

#### Zeta potential method

Table 2 shows the zeta potential value of every nanofluid. According to the literature, all samples has good stability, which indicates with zeta potential value greater than |40|^34^. The negative values indicate that all the particles are negatively charged in suspension. The AO/SDBS sample has a zeta potential value of − 52.85 mV. This value is in accordance with the literature^35^. The AO/CMC and AO/TA/CMC have excellent stability with zeta potential value of − 72.29 and − 60.53 mV, respectively. Compared to AO/SDBS, the AO/CMC zeta potential value was improved by 37%. This significant improvement is attributed to CMC acting as a stabilizing agent in the nanofluid, providing stronger electrostatic epulsion between particles and potentially higher stability in suspension.

**Table 2:** Zeta potential value of all nanofluid samples.

Sample	Zeta Potential (mV)
AO/SDBS	− 52.85 ± 0.56
AO/TA	− 51.88 ± 1.09
AO/CMC	− 72.29 ± 0.65
AO/TA/CMC	− 60.53 ± 0.28

CMC provides stabilization to the nanoparticles through the charges present in its molecular structure, leading to electrostatic repulsion^20^. These charges impart a negative charge to the Al_2_O_3_, preventing the particles from approaching each other and thereby enhancing stability^36^. The AO/TA sample also has high zeta potential value, which is close to AO/SDBS sample. This may prove that the TA provides steric repulsion to Al_2_O_3_ that similar to SDBS_._ This finding aligns to the previous work research related to using TA for nanofluid stabilization^14^.

However, the AO/TA/CMC did not result in better stability than AO/CMC. This observation may be attributed to the hypothesis that TA might not function optimally in the high electrostatic or charge stabilization region^37^. This study was conducted at a constant room temperature. However, in practical applications, nanofluids are often subjected to fluctuating temperatures that can affect their stability. Temperature variations can alter the solubility of the modifying agents, the viscosity of the fluid, and the kinetic energy of the nanoparticles, potentially leading to agglomeration or changes in zeta potential^28^. Moreover, this study does not investigate the effects of pH and shear rate on the nanofluid system. Both pH and shear rate are critical in real-world applications; pH can influence the surface charge and hence the zeta potential of nanoparticles, while shear rate can affect particle distribution and alignment due to fluid dynamics^28^. Therefore, future research should extend the scope to include these variables to fully characterize the nanofluids under a broader range of conditions.

### Tribological properties analysis

#### Friction coefficient

**Figure 5:**
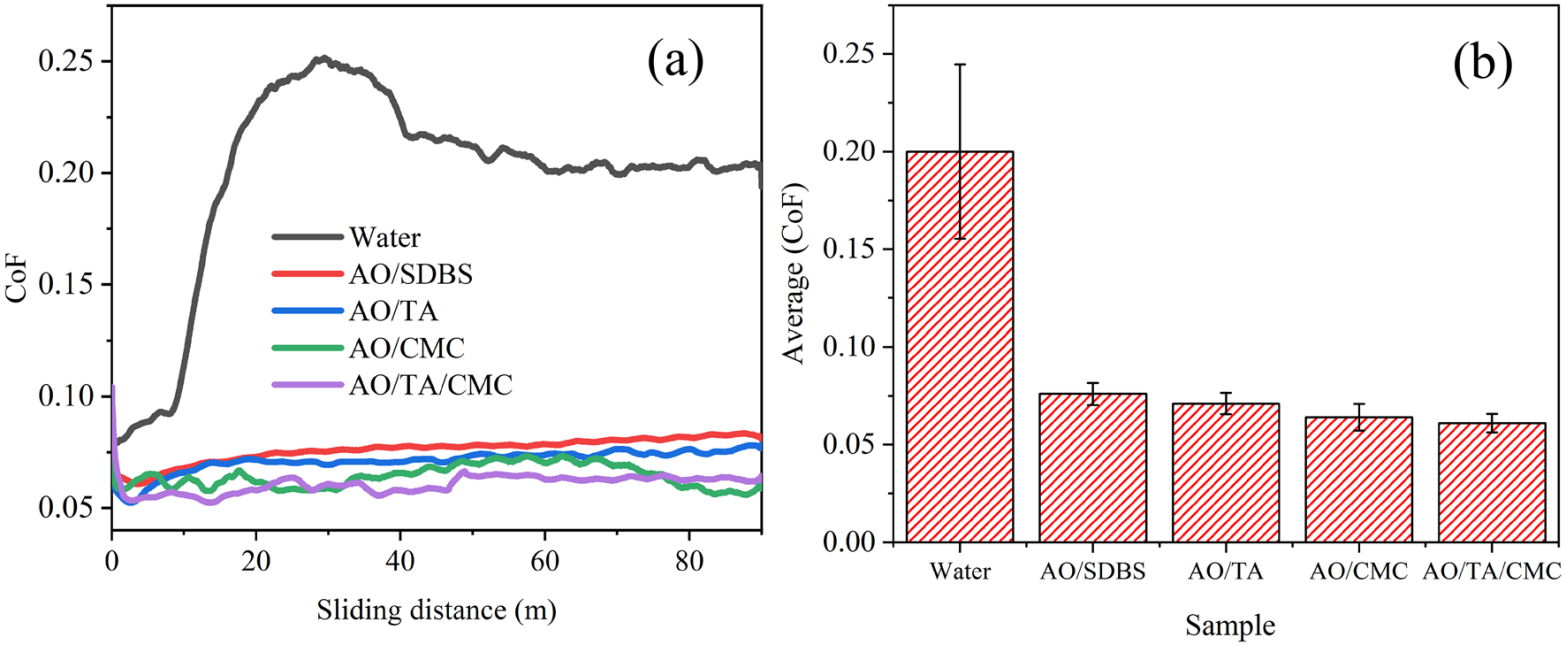
Ball-on-disk tribological behavior of different nanofluid: CoF-sliding distance graph (**a**), and average CoF (**b**).

Figure 5a,b display the CoF of the lubricated disk for the total duration and the average CoF value, respectively. It can be seen in Fig. 5a that all samples exhibit better CoF compared to water because of the presence of Al_2_O_3_ particles^38^. The disk lubricated with AO/SDBS has relatively stable CoF, almost three times lower than neat water. This finding is attributed to the role of spherical Al_2_O_3_ particles, providing a rolling effect that transforms sliding friction into rolling friction^6^. The AO/TA sample has an average CoF of 0.071, which is slightly lower than AO/SDBS.

In the previous work, Uncaria gambir extract (UGE), which has about 50% tannic, was found to provide a more stable CoF profile compared to those without UGE^18,39,40^. Another recent work also reported that adding TA improved or reduced the CoF^41,42^. On the other hand, AO/CMC exhibited an average CoF of 0.064. This is due to the CMC properties that can replenish the base fluid's degraded viscosity and form thicker films between sliding surface, resulting in lower friction^17^.

Notably, the AO/CMC CoF fluctuates more than others at the first 20 m of sliding distance, indicating a stick–slip effect^43^. Incorporating both TA and CMC results in the lowest CoF, which is 19.7% lower compared to the AO/SDBS sample. This may be attributed to the phenolic compounds of TA that could react with the surface of the disk, forming a protective layer that increases the film thickness. On the other hand, the CMC provides electrostatic stabilization mechanisms that effectively prevent nanoparticle aggregation by reducing the van der Waals force force, ensuring an even distribution and reducing CoF^20,37,44^.

#### Wear

**Figure 6:**
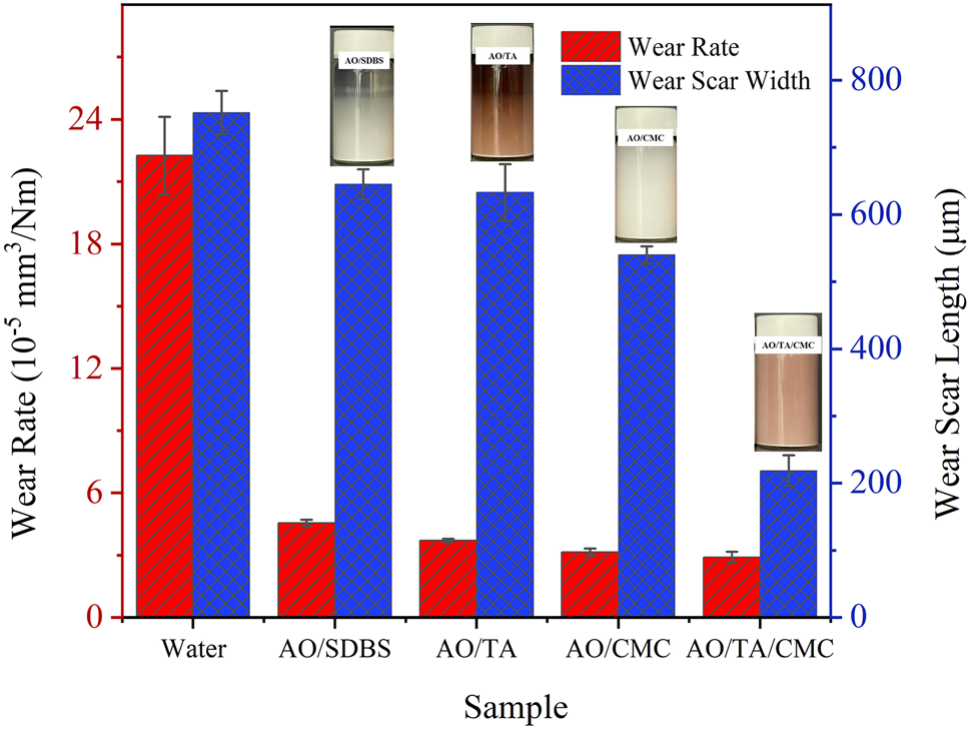
Wear rate and wear scar width of disk lubricated with nanofluids.

**Figure 7:**
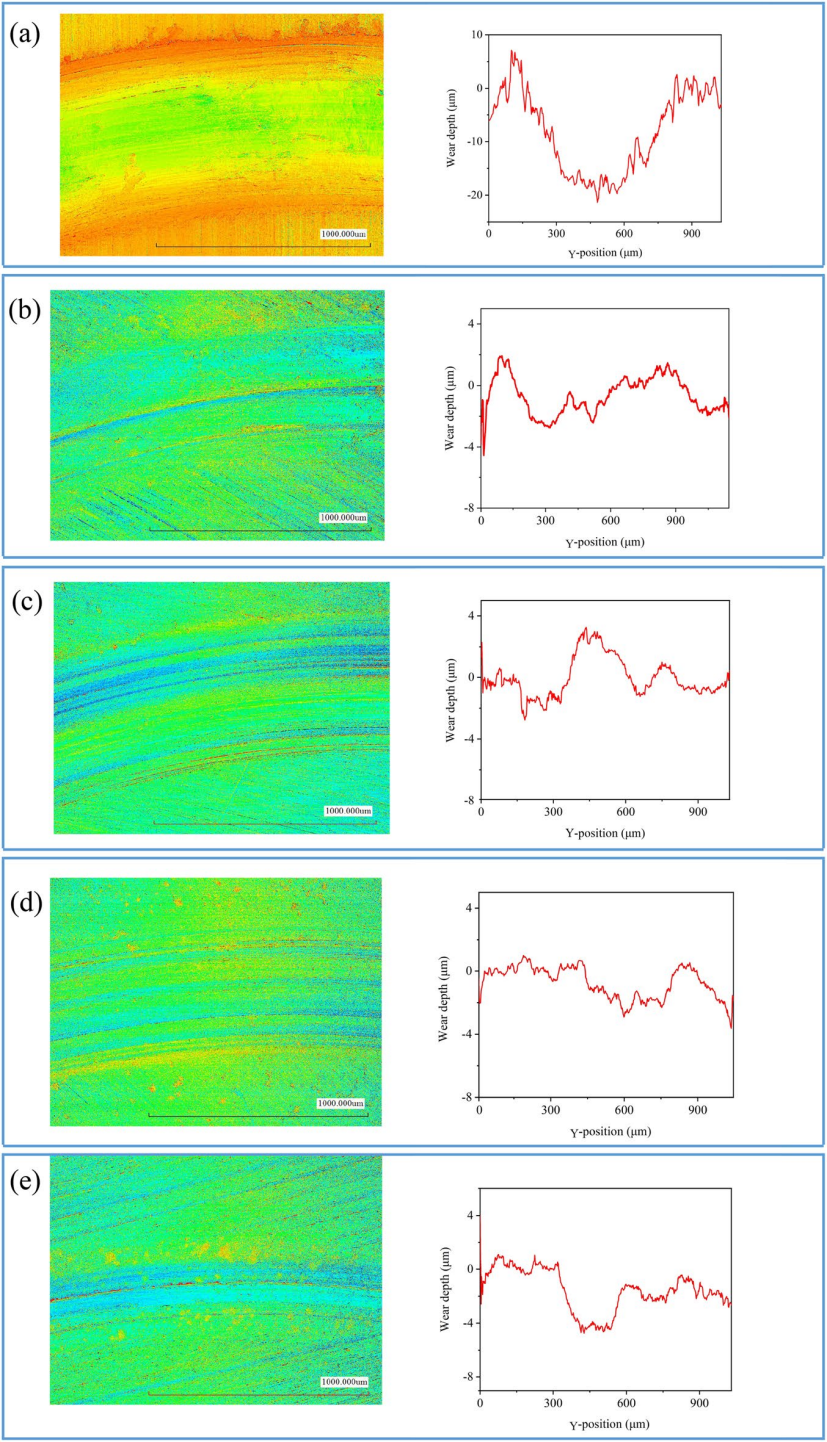
Surface topography and cross-section depth profile of wear scar on the disk lubricated by water (**a**), AO/SDBS (**b**), AO/TA (**c**), AO/CMC (**d**), and AO/TA/CMC (**e**).

Figure 6 displays the disk's wear rate and wear scar width after undergoing a ball-on-disk tribological test. The surface topography and cross-section depth profile of the wear scar on the disk lubricated with the nanofluid samples are presented in Fig. 7. The wear rate of the AO/SDBS was improved by 5 times compared to water. Adding TA and CMC decreased the wear rate by 19% and 32%, respectively. The disk lubricated with AO/TA/CMC nanofluid exhibited the lowest wear rate, which was 59% lower than that of the disk lubricated with the AO/SDBS sample. These results are consistent with the CoF values, which show an improvement trend following the addition of additives.

On the other hand, the wear scar width of the rubbed disk also decreased with the increase in sample stability. As seen in Fig. 7, the disk lubricated with neat water has a maximum wear depth of about 18 μm, which is far deeper compared to that lubricated with nanofluid. The AO/SDBS sample exhibits a low depth with a maximum depth of about 2.6 μm. However, it can be seen that the wear scar width is higher compared to disk lubricated with other nanofluids, which explains the high wear rate compared to the disk lubricated with others. This is probably attributable to poorly stabilized AO/SDBS sample that lead to the agglomeration of nanoparticles. When applied as a lubricant, these agglomerated nanoparticles distribute unevenly, causing enhanced surface roughness and resulting in increased friction.

After incorporating TA and CMC, the wear rate and wear scar width are lower compared to the disk lubricated with AO/SDBS. It also can be seen that it has less furrow or relatively low roughness. The reasons for this phenomenon will be further discussed in the following section. Interestingly, although the wear scar width becomes smaller after adding TA and CMC, the wear depth is much deeper than that lubricated with other nanofluids. It can be inferred that the presence of nanoparticles can prevent the direct contact of friction surfaces but may also behave as wear particles, that aggravate abrasive wear at the same time. This phenomenon is similar to one literature that also works using nanofluid^38,45^.

**Figure 8:**
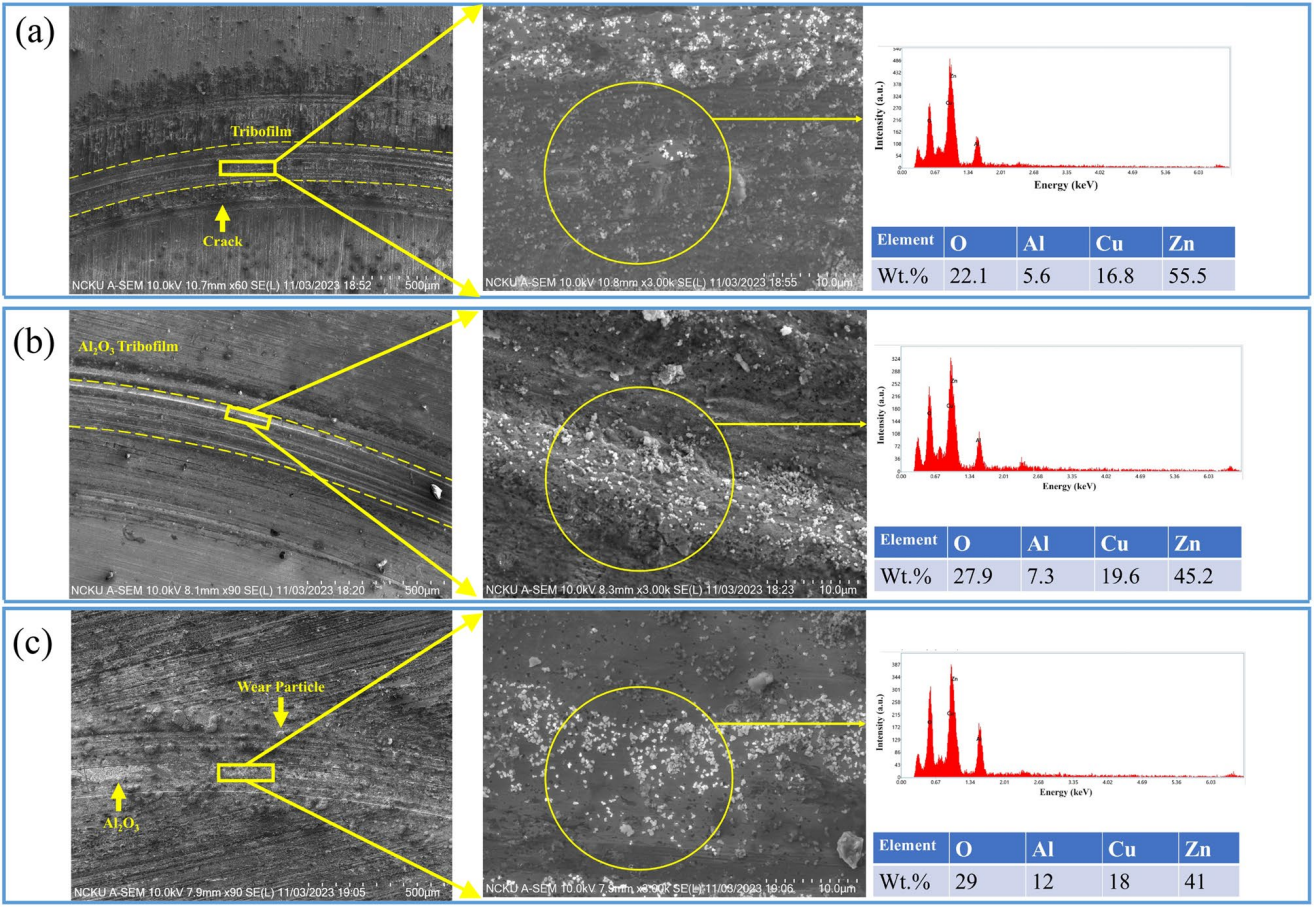
The SEM–EDS analysis of the disk surface lubricated by AO/TA (**a**), AO/CMC (**b**), and AO/TA/CMC (**c**).

To gain a deeper understanding of TA and CMC behavior in tribological properties, the worn disk surfaces lubricated with AO/TA, AO/CMC, and AO/TA/CMC were investigated using scanning electron microscopy (SEM), as illustrated in Fig. 8. The SEM image of the disk lubricated with AO/TA (Fig. 8a) reveals the presence of cracks on the worn surface, likely due to lower stability compared to the other nanofluids. This uneven stability may result in an inconsistent distribution of aluminum, contributing to localized wear. This observation is supported by the EDS analysis, confirming that only 5.6% of Al is detected.

For the AO/CMC (Fig. 8b), interestingly, the disks has a notably smooth surface. Notably, the presence of a visible tribofilm and Al_2_O_3_ particles indicates a mending effect, where the nanofluid contributes to surface repair during tribological contact^46^. The observed smooth surface consistent with the excellent stability of the AO/CMC nanofluid. The stability likely facilitates an even distribution of nanoparticles, ensuring effective lubrication across the surface^47^. Finally, AO/TA/CMC exhibits the smallest wear scar among the three samples. It can be observed that the Al_2_O_3_ nanoparticles are distributed evenly without any clusters detected. This is supported by the highest concentration of Al elements detected from the EDS (12%).

### Corrosion performance analysis

**Figure 9:**
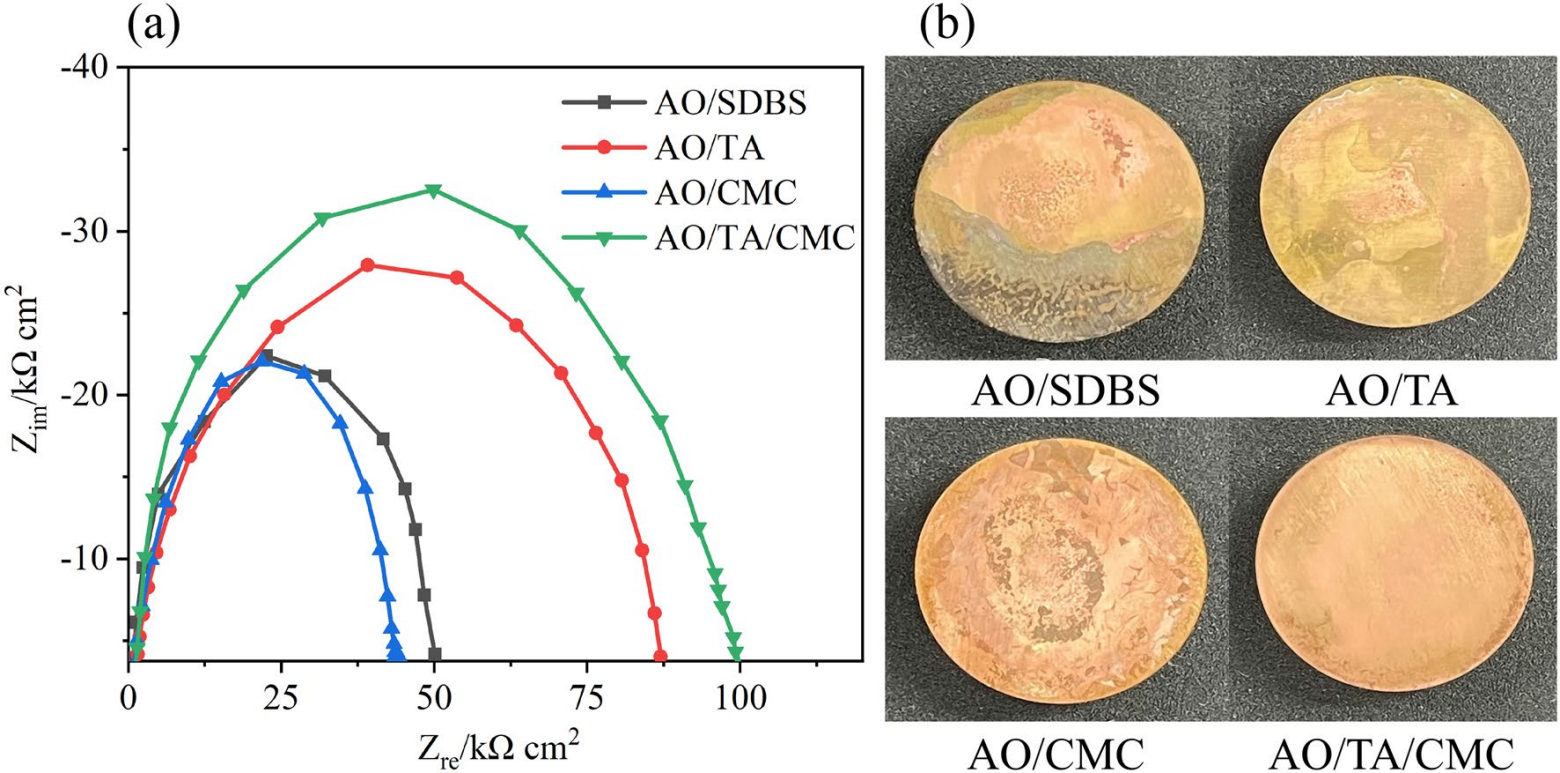
The EIS diagram (**a**), and Figures of Cu–Zn alloy surface after 3 days immersion (**b**).

**Figure 10:**
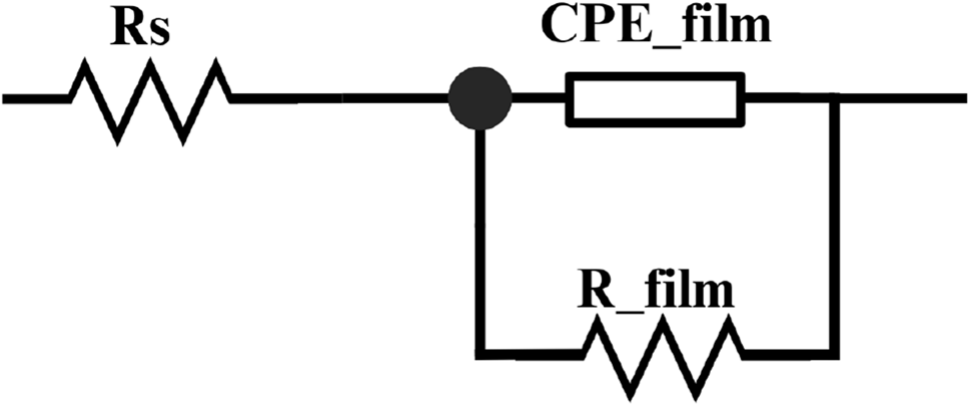
EIS equivalent circuit diagram.

Figure 9a shows the EIS diagram or Nyquist plots of the Cu–Zn alloy immersed in every nanofluid sample, and Fig. 9b exhibits its surface after being immersed for 3 days. Figure 10 illustrates the equivalent circuit for fitting EIS. R_s_ represents the resistance of the solution; CPE_film is the capacitance of the film; R_film is the film resistance between the Cu–Zn alloy and solution that originated from the nanofluid. CPE was used in this study as opposed to the capacitance in the traditional equivalent circuit model as there was an uneven current potential distribution; CPE would be more accurate according to previous studies^48^.

The EIS fitting results are displayed in Table 3. It is proven that the presence of different additives in the nanofluid affects both R_s_ and R_film. R_s_ values are relatively consistent across all samples. However, there is notable variation in the R_film values, with the AO/TA/CMC combination exhibiting the highest resistance at 99.2 Ω, which was 97.6% higher compared to AO/SDBS. This implies that the synergistic effect of TA and CME in the nanofluid creates a more effective barrier against corrosion compared to others.

**Table 3:** EIS fitting results of Cu–Zn allow disk continuously immersed in nanofluid for 3 days.

Sample	R_s_	R_film_	CPE_film-P	CPE_film-T
AO/SDBS	22.3	50.2	0.82955	1.60 × 10^–9^
AO/TA	27.9	87.1	0.80293	1.79 × 10^–9^
AO/CMC	22.1	43.4	0.81265	1.65 × 10^–9^
AO/TA/CMC	32	99.2	0.79321	1.43 × 10^–9^

The CPE_film-P values are close to unity across all samples, suggesting capacitive behavior with minor deviations. The CPE_film-T values, which are indicative of the time constant of the film capacitance, are lowest for the AO/TA/CME combination, aligning with the highest R_film observed, further confirming the efficacy of this particular nanofluid combination in enhancing the protective film's properties.

Table 4 exhibited the weight loss of Cu–Zn alloy disk immersed in the nanofluid combined with NaCl solution. Comparing these results with the EIS data from Table 3, a clear correlation emerges. Samples with higher R_film values, indicating greater resistance to charge transfer and thus better protection, correspond to lower average weight loss rates. Specifically, the AO/TA/CME sample, which has the highest R_film, also shows the lowest weight loss rate of 0.102 g/(m^2^h). Figure 9b shows the surface of the disk after immersion for 3 days. Lots of corrosion pits were detected on the disk surface after it immersed in AO/SDBS and salt water, indicating that high corrosive reactions occurred. When immersed in TA, only slight corrosion pits and the surfaces were relatively smooth. Immersing with AO/TA/CMC provides smoothest disk surface. This correlation confirms the consistency and validity of the corrosion measurements obtained by both EIS and weight loss methods.

**Table 4:** Weight loss results in solutions with various nanofluid samples after 3 days immersion.

Sample	Average v (g (m^2^h))
AO/SDBS	0.270 ± 0.004
AO/TA	0.105 ± 0.002
AO/CMC	0.216 ± 0.002
AO/TA/CMC	0.102 ± 0.005

This outcome is a synergistic effect of the combined presence of TA and CMC, that provide enhanced corrosion protection. TA, known for its metal-chelating and antioxidant properties, likely forms a protective layer on the alloy surface, which contributes to its corrosion inhibition potential^49,50^. The incorporation of CMC not only acts as stabilization but also introduces a corrosion protection capability. This dispersion not only enhances the physical barrier formed by TA but also imparts a negative charge to the alloy surface, creating electrostatic repulsion between particles. Incorporating TA and CMC results in a more effective corrosion inhibition.

The differences in impedance between the samples can also be influenced by the dispersion stability of the nanofluids, as reflected by their zeta potential values. The increased stability, as suggested by the more negative zeta potential in the AO/CMC and AO/TA/CMC sample, leads to a lower tendency for particle aggregation, which could contribute to an increase in charge transfer resistance.

### TA and CMC mechanism as surfactant, anti-corrosion additives, and friction modifier

**Figure 11:**
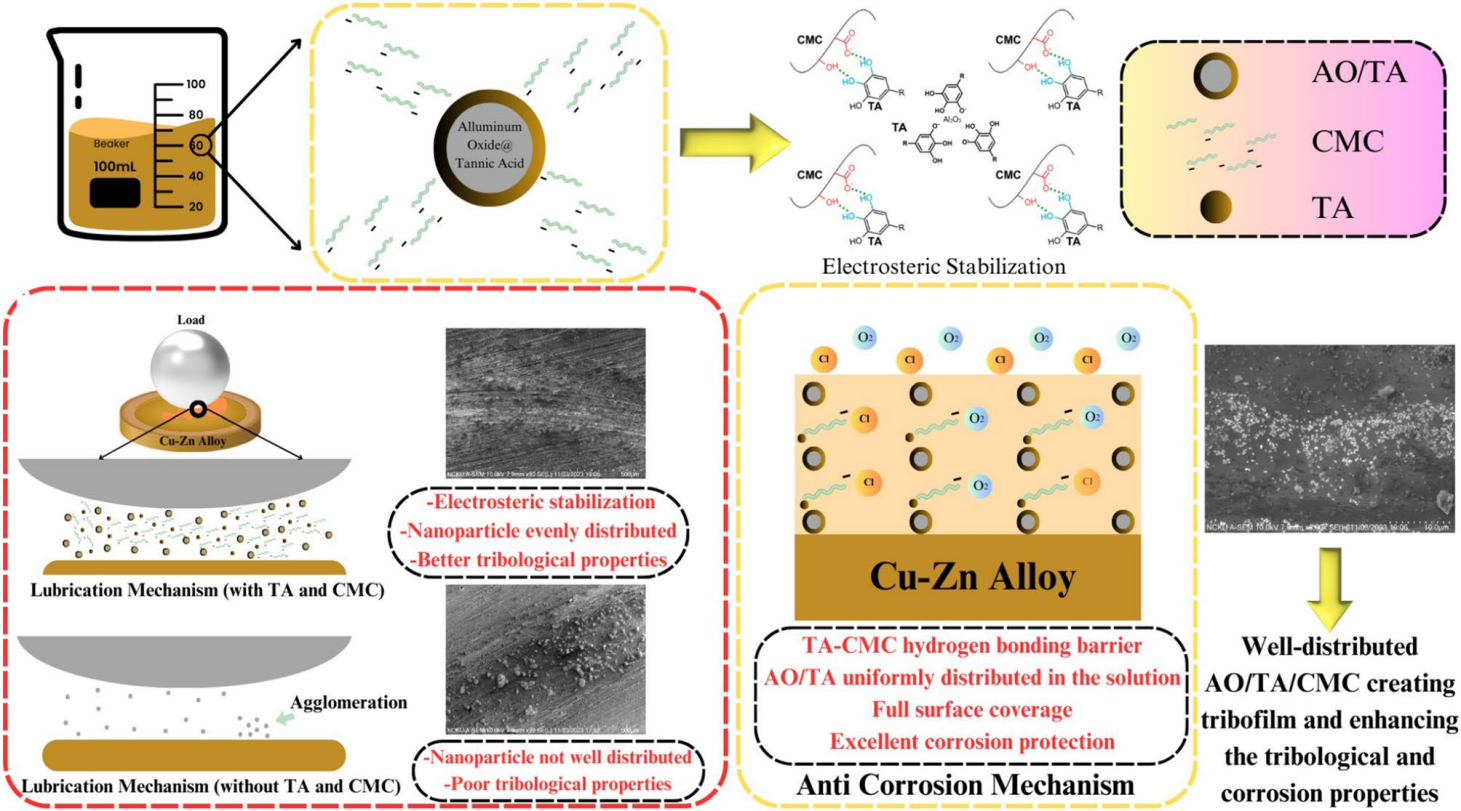
Schematic diagrams of lubrication and corrosion mechanism.

Based on the investigation, AO/TA/CMC is the best sample due to its excellent, stability and tribocorrosion properties. Lubrication and corrosion mechanism of AO/TA/CMC are proposed and illustrated in Fig. 11^51^. Al_2_O_3_ nanoparticles contribute solid lubrication properties, forming a barrier that prevent metal-to-metal surfaces contact, reducing friction and wear^52^. Incorporating both TA and CMC introduces a sophisticated interplay of tribological mechanisms.

CMC imparts electrostatic repulsion to Al_2_O_3_ nanoparticles. This electrostatic interaction ensures dispersion stability, preventing agglomeration and maintaining an even distribution of nanoparticles across the lubricated surfaces, resulting in well-ordered protective film^53^. Moreover, CMC also acts as a thickener and can enhance the viscosity of the base fluid. This effect offers a dual advantage since increased viscosity is associated with improved stability and tribological properties^53,54^. On the other hand, albeit cannot support CMC as stabilizer, TA is introduced primarily for corrosion protection. Its polyphenolic nature forms a protective layer on metal surfaces, mitigating corrosive reactions. This protective layer acts as a barrier, preventing the degradation of metal components exposed to corrosive environments.

The combined action of CMC and TA yields a synergetic effect on friction reduction. CMC, acting as a thickener, contributes to viscosity control, reducing friction in high-shear conditions^55^. The hydroxyl groups in TA and the oxygen-containing groups in CMC can participate in hydrogen bonding interactions^51^. This bonding may help enhance the adhesion and cohesion of the protective layer, making it more resistant to environmental factors and improving its ability to provide corrosion protection.

## Conclusions

In conclusion, we have successfully prepared and demonstrated the effectiveness of TA and CMC in enhancing Al_2_O_3_ nanofluid stability and tribocorrosion properties. When compared to the sample without TA or CMC, the lubricated disk exhibits lower coefficients of friction (CoF), lower wear rates, and improved corrosion resistance. The addition of both TA and CMC provides excellent stability and the best CoF improvements, with a 59% reduction compared to the sample without TA and CMC. It also shows the highest corrosion resistance value, with a 97.6% higher resistance and 62% lower mass loss compared to AO/SDBS. TA acts as a corrosion inhibitor due to its polyphenolic nature, forming a protective layer on metal surfaces. On the other hand, CMC provides electrostatic repulsion that counters the attractive forces between nanoparticles. These results indicate that TA and CMC show promising potential as water-based nanofluid additives, offering insights for designing high-performance and environmentally friendly water-based lubricants or coolants for machining applications.

The future potential of TA and CMC holds a promising role as effective nanofluid additives for lubricant or coolant applications, primarily for the enhancement of stability and corrosion prevention. This current research highlights the positive impact of combining TA and CMC into Al_2_O_3_ nanofluid. This synergistic combination has demonstrated improvements in stability, tribological, and corrosion properties, contributing to prolonged shelf life and sustained performance.

However, to fully unlock the potential of this nanofluid system, further research is needed. Specifically, investigating the optimal concentration levels for Al_2_O_3_, TA, and CMC in the nanofluid is crucial. Understanding the intricate balance among these components will not only maximize stability, tribological properties, and corrosion resistance but also pave the way for enhanced thermal conductivity and other desirable properties. Furthermore, investigating other properties such as pH, viscosity, stability at high temperatures, electrical properties, and optical properties would be very interesting to conduct. Moreover, it is also necessary to do comparative study with other types of nanofluids or additives that have been available recently. This exploration holds the key to refining the nanofluid formulation for diverse applications, ranging from heat transfer fluids to advanced cooling systems, thereby advancing the frontier of nanofluid technology.

## Data Availability

All data generated or analyzed during this study are included in this published article.
